# Corticosteroid-depending effects on peripheral immune cell subsets vary according to disease modifying strategies in multiple sclerosis

**DOI:** 10.3389/fimmu.2024.1404316

**Published:** 2024-06-13

**Authors:** Lena Höpner, Undine Proschmann, Hernan Inojosa, Tjalf Ziemssen, Katja Akgün

**Affiliations:** Center of Clinical Neuroscience, Department of Neurology, Faculty of Medicine and University Hospital Carl Gustav Carus, Technical University Dresden, Dresden, Germany

**Keywords:** multiple sclerosis, methylprednisolone treatment, immunophenotyping, disease modifying therapies, relapse

## Abstract

**Background:**

The primary treatment for acute relapses in multiple sclerosis (MS) is the intravenous administration of high-dose methylprednisolone (IVMP). However, the mechanisms through which corticosteroid treatment impacts acute neuroinflammation in people with MS (pwMS) remain not fully understood. In particular, the changes induced by glucocorticoids (GCs) on cells of the innate immune system and the differences between patients with distinct immunotherapies have received little attention to date.

**Methods:**

We conducted immunophenotyping using flow cytometry on peripheral blood mononuclear cells of pwMS who received IVMP treatment during a relapse. We compared the impact of an IVMP treatment on a broad variety of immune cell subsets within three groups: twelve patients who were treatment-naïve to disease modifying therapies (wDMT) to ten patients on platform therapies (PT) and eighteen patients on fingolimod therapy (FTY).

**Results:**

We observed pronounced interindividual short- and intermediate-term effects of IVMP on distinct immune cells subsets. In addition to the well-documented decrease in T-helper cells (Th cells), we detected significant alterations after the first IVMP infusion within the innate immune response among neutrophil, eosinophil and basophil granulocytes, monocytes and plasmacytoid dendritic cells (pDCs). When comparing patients wDMT to the PT and FTY cohorts, we found that IVMP had a similar impact on innate immune cells across all treatment groups. However, we did not observe a significant further decline in T lymphocyte counts during IVMP in patients with pre-existing lymphopenia under FTY treatment. Although T cell apoptosis is considered the main mechanism of action of GCs, patients with FTY still reported symptom improvement following IVMP treatment.

**Conclusion:**

In addition to T cell suppression, our data suggests that further immunoregulatory mechanisms of GC, particularly on cells of the innate immune response, are of greater significance than previously understood. Due to the regulation of the adaptive immune cells by DMTs, the impact of GC on these cells varies depending on the underlying DMT. Additional studies involving larger cohorts and cerebrospinal fluid samples are necessary to gain a deeper understanding of the immune response to GC in pwMS with different DMTs during relapse to define and explain differences in clinical response profiles.

## 1 Introduction

Multiple Sclerosis (MS) is a chronic inflammatory demyelinating autoimmune disease of the central nervous system (CNS) driven by T-cells, B-cells and cells of the innate immune system (1). A relapsing disease course is the most common course, especially in younger patients (2). The primary treatment for acute relapse is the intravenous administration of 500–1.000mg of methylprednisolone (MP) for three or five consecutive days, which leads in most patients to the amelioration of symptoms (3–5).

MP is widely used in the treatment of inflammatory and autoimmune diseases. Its immunosuppressive effect is mainly mediated by the interaction with the glucocorticoid-receptor (GR), which can lead to the activation or suppression of immunoregulatory genes (6). In pwMS, the suppression of T-cells is considered a key mechanism contributing to the therapeutic effect of IVMP therapy (6, 7). However, the precise role of other immune cells and their specific immunoregulatory actions in resolving acute neuroinflammation in pwMS remain yet not completely understood (8, 9).

In addition to corticosteroid therapy for acute relapses, DMTs play a crucial role in preventing further disease activity and future relapses, with various therapeutic options available (10, 11). Whereas interferon-β, dimethyl fumarate and teriflunomide are used for less active disease courses, sphingosine-1-phosphate (S1P) receptor modulators, among other options, are approved as treatment for more active relapsing MS. They prevent the egress of lymphocytes from the lymphoid tissues by inducing receptor internalization and degradation (12, 13).

Besides clinical characteristics and magnetic resonance imaging, molecular biomarkers in cerebrospinal fluid (CSF) play a significant role in the diagnosis and monitoring of MS (14). While immunoprofiling of peripheral blood mononuclear cells (PBMC) has been proposed as a potential biomarker for disease activity and treatment response, studies with real world data that include the cells of the innate immune system are lacking (15, 16).

In this study, we investigated peripheral immune cell subsets in pwMS during acute relapse based on real world data. We examined longitudinal changes under IVMP therapy, following our pilot study which had already revealed interesting changes in immune cell populations after premedication with MP before alemtuzumab therapy (17). Our objective was to comprehensively understand the ex-vivo effects of corticosteroid therapy on the immune cells of pwMS and according to the use of distinct DMTs. By identifying immunological patterns at relapse and during MP treatment, we aim to provide further insights into the response profile to GC treatment in pwMS with different underlying DMTs.

## 2 Materials and methods

### 2.1 Participants and study approval

The study was conducted at the MS center at Carl Gustav Carus University Hospital, Technical University Dresden, Germany. Patients diagnosed with relapsing MS, according to the 2017 revised McDonald criteria, who presented with an acute relapse starting on MP treatment, were consecutively recruited from October 2016 to May 2019 (18). Relapse was characterized by the occurrence of MS typical symptoms lasting at least 24 hours, with a 30-day interval since the last relapse and in absence of fever or infection (18). Medical history and clinical parameters, including changes in the Expanded Disability Status Score (EDSS), were evaluated by specialized neurologists (**Table 1**) (19). The study cohort consisted of 58 patients with different DMT strategies. Due to the wide dispersion of data within the individual therapy groups, we focused on the largest treatment cohorts and excluded 18 patients receiving natalizumab, ocrelizumab, daclizumab or alemtuzumab treatment. For our analysis, we selected patients without a disease modifying therapy (wDMT, n=12). In a second step, we compared the immune cell subsets of this group to two other cohorts: patients on platform therapy (PT, n=10), including two patients on Peginterferon beta-1a (Plegridy^®^, Biogen Cambridge, MA, USA), six on dimethyl fumarate (Tecfidera^®^, Biogen Cambridge, MA, USA) and two on teriflunomide (Aubagio^®^, Sanofi, Paris, France); and patients with a highly active disease course on fingolimod (FTY, n=18) (Gilenya^®^, Novartis, Basel, Switzerland). The study was approved by the institutional review board of the University Hospital of Dresden (EK348092014, EK35012021). Written informed consent was obtained by all patients.

**Table 1:** Demographics and clinical characteristics of patients with relapse.

Characteristics	All (*n*=40)	wDMT (*n*=12)	PT ^1^ (*n*=10)	FTY (*n*=18)
Sex, *n* (%)				
Female	29 (72%)	11 (92%)	9 (90%)	9 (50%)
Male	11 (28%)	1 (8%)	1 (10%)	9 (50%)
Age, years at relapse (mean, SD)	44.83 ± 12.97	45.42 ± 17.95	40.80 ± 12.83	46.67 ± 8.79
Disease duration, years at relapse (median, IQR)	9.15 (5.20–19.25)	10.70 (3.10–20.75)	5.80 (3.25–6.95)	15.00 (9.23–20.50)
Disease course, *n* (%)				
RRMS	37 (92%)	9 (75%)	10 (100%)	18 (100%)
SPMS	3 (8%)	3 (25%)	0 (0%)	0 (0%)
EDSS at relapse (median, IQR)	4 (2–5)	4 (2–5)	2 (2–3)	4 (3–5.5)
Difference in EDSS^2^ (median, IQR)	0 (0–1)	1 (0.5–1.5)	0 (0–0)	0 (0–0.25)
Remission^3^, *n* (%)				
Yes	19 (47%)	4 (33%)	4 (40%)	11 (61%)
Partially	12 (30%)	6 (50%)	4 (40%)	2 (11%)
No	9 (23%)	2 (17%)	2 (20%)	5 (28%)
Methylprednisolon, i.v., *n* (%)				
1000mg	39 (97%)	12 (100%)	10 (100%)	17 (94%)
500mg	1 (3%)	0 (0%)	0 (0%)	1 (6%)

EDSS, expanded disability status scale; FTY, fingolimod; IQR, interquartile range; i.v.: intravenous; n, patient count; PT, platform therapy; RRMS, relapsing-remitting multiple sclerosis; SD, standard deviation; SPMS, secondary progressive multiple sclerosis; wDMT, without disease-modifying-therapy.

^1^platform therapy includes: Interferon (n=2), dimethyl fumarate (n=6), teriflunomide (n=2).

^2^difference between EDSS at relapse and a previous EDSS at steady state.

^3^subjective assessment by patients.

### 2.2 Infusion protocol and blood sampling

Patients underwent IVMP treatment based on a standardized infusion protocol used in our MS Center. A daily dose of 1000mg (one patient with 500mg) of intravenous MP (Urbason^®^ solubile forte) was administered for three or five consecutive days. In one specific case, a patient received a daily dose of 500mg. Prior to treatment, acute infections or contraindications to therapy were ruled out for safety. Heparinized blood samples were collected at relapse before the start of treatment (baseline, relapse, R), 24 hours after the first infusion (T1), 24h after the administration of the second infusion (T2) as well as two weeks (T3) and two months (T4) after MP therapy. PBMC were isolated by Ficoll-Hypaque (Biochrom, Berlin, Germany) density centrifugation. Cells were frozen using fetal calf serum (FCS, Gibco) with 10% dimethylsulfoxid (DMSO) (Sigma-Aldrich, St. Louis, US-MO) and controlled rate freezing containers (Nalgene Nunc Int., Rochester, US-NY). Afterwards cells were cryo-preserved at -80°C until collective analysis was performed.

### 2.3 Routine blood analysis

Standardized blood testing was performed for routine complete blood cell count parameters at the Institute of Clinical Chemistry and Laboratory Medicine, Carl Gustav Carus University Hospital, Technical University Dresden, Germany. The institute complies with standards required by DIN-EN-ISO-15189 for medical laboratories.

### 2.4 Immune cell phenotyping by fluorescence-activated cell sorting

Frozen PBMC were thawed and washed with fluorescence-activated cell sorting (FACS) buffer (phosphate buffered saline, 0.2% fetal calf serum, 0.02% sodium azide) before being incubated with the viability marker Zombie Green (Biolegend, San Diego, CA, USA). Subpopulations of T cells, B cells, natural killer (NK) cells and antigen-presenting cells (APC) were characterized by surface staining with a combination of fluorochrome-conjugated monoclonal antibodies against anti cluster of differentiation (CD) 5, anti-CD8, anti-CD16, anti-CD56, anti-CD80, anti-CD83, anti-CD86, anti-CD94, anti-BDCA1, anti-BDCA2, anti-BDCA4, anti-HLADR (Biolegend, San Diego, CA, USA); anti-CD3, anti-CD4, anti-CD10, anti-CD14, anti-CD19, anti-CD25, anti-CD27, anti-CD38, anti-CD40, anti-CD45RA, anti-CD45RO, anti-CD69, anti-CD123, anti-CD138 (BD Biosciences, San Jose, CA, USA); anti-MDC8, anti-BDCA3 (Miltenyi Biotec, Bergisch-Gladbach, Germany) according to the manufacturer´s instructions. Isotype-matched irrelevant antibodies (Biolegend, San Diego, CA, USA; BD Biosciences, San Jose, CA, USA) were used as negative controls for unspecific antibody binding. For intracytoplasmic cytokine measurement PBMC were washed and resuspended in culture medium consisting of RPMI 1640 (Biochrom, Berlin, Germany), 5% human AB serum (CC pro, Neustadt, Germany), 2 mM L-glutamine, 100 U/ml penicillin and 100 µg/ml streptomycin (Biochrom, Berlin, Germany). Afterwards the cells were stimulated with 10ng/ml phorbol myristate acetate (PMA, Sigma-Aldrich, St. Louis, US-MO), 1µg/ml ionomycin (Sigma-Aldrich, St. Louis, US-MO) and 0.2 µM Monensin (Biomol, Hamburg, Germany) and then incubated for 6 hours at 37°C and 5% CO<sub>2</sub>. Before staining, cells were fixed with 4% paraformaldehyde (PFA, Merck, Darmstadt, Germany) and permeabilized with 0.1% saponin (Sigma-Aldrich, St. Louis, US-MO). For intracellular labeling the fluorescence-marked antibodies anti-interferon-gamma (INFγ), anti-interleukin (IL)-17A (Biolegend, San Diego, CA, USA), anti-FoxP3 (Miltenyi Biotec, Bergisch-Gladbach, Germany) and isotype-matched irrelevant antibodies (BD Biosciences, San Jose, CA, USA) were used. Samples were run on a LSR-Fortessa flow cytometer (BD Biosciences, San Jose, CA, USA). FACS-Diva Software (BD Biosciences, San Jose, CA, USA) was used for data analysis.

### 2.5 Statistical analysis

For the characterization of the study population and relapse characteristics quantitative variables were presented as mean and standard deviation (SD) whereas categorical variables were shown as absolute (n) and relative frequencies (%). Prior to analysis, the immunological data set was tested for normal distribution using quantile-quantile plots and histograms and confirmed with the Shapiro-Wilk test. For the longitudinally analysis, Generalized Linear Mixed Models were applied, with Bonferroni correction for pairwise tests as appropriate to compare cell subsets over time during and after IVMP. In case of right-skewed distribution patterns, gamma distribution and log-link function transformation were implemented. Differences in mean values among DMT groups were evaluated using the Kruskal-Wallis-Test with subsequent pairwise *post-hoc* comparisons conducted with the Dunn-Bonferroni test. Few cases of missing data were handled by listwise deletion. Values of p were considered statistically significant as follows: * p<0.05, ** p<0.01, *** p<0.001. Statistical analysis was performed using the IBM SPSS Software (Version 27.0; IBM Corporation, Armonk, NY, USA). Graphs were created with GraphPad Prism8 (GraphPad Software, San Diego, CA, USA).

## 3 Results

### 3.1 Clinical characteristics of patients with relapse

The study cohort consisted of 40 pwMS, with an average age of 44.83 ± 12.97 years. Among them, 29 (72%) were female and 37 (92%) were diagnosed with RRMS. The cohort comprised twelve patients wDMT, ten patients with PT and eighteen patients treated with FTY (**Table 1**). Patients treated with FTY tended to have the longest disease duration (**Table 1**). However, changes in the EDSS during relapse compared to a previous steady state, were more pronounced in the untreated cohort, followed by the FTY cohort and were lowest in the PT cohort (**Table 1**). All patients, except one, received a daily dose of 1000mg IVMP for three or five consecutive days. In the case of one patient of the FTY cohort, a daily dose of 500mg for three days was administered because of a history of myopathic symptoms during statin therapy. Two weeks after completing the IVMP treatment, the majority of patients in all three groups reported either an improvement or at least partial improvement of their initial symptoms. No effect was noted by two patients (17%) wDMT, two patients (20%) with PT and five patients (28%) with FTY. The classification was based on the subjective perception of the patients. In the overall cohort of all study participants, only one patient (69 years old, SPMS, wDMT) had a MP-associated infection after a second cortisone cycle, where she developed urosepsis. Data collection is incomplete for at least one time point during follow-up for *n*=3 in the treatment-naïve cohort, *n*=2 in the PT cohort and *n*=8 in the FTY cohort. The overall rate of missed visits was 8%.

### 3.2 Effect of corticosteroid treatment on peripheral immune cell subsets in patients wDMT

To explore the impact of an IVMP therapy on the immune cells of MS patients experiencing relapse, we initially analyzed a group of twelve treatment naïve patients.

**Figure 1:**
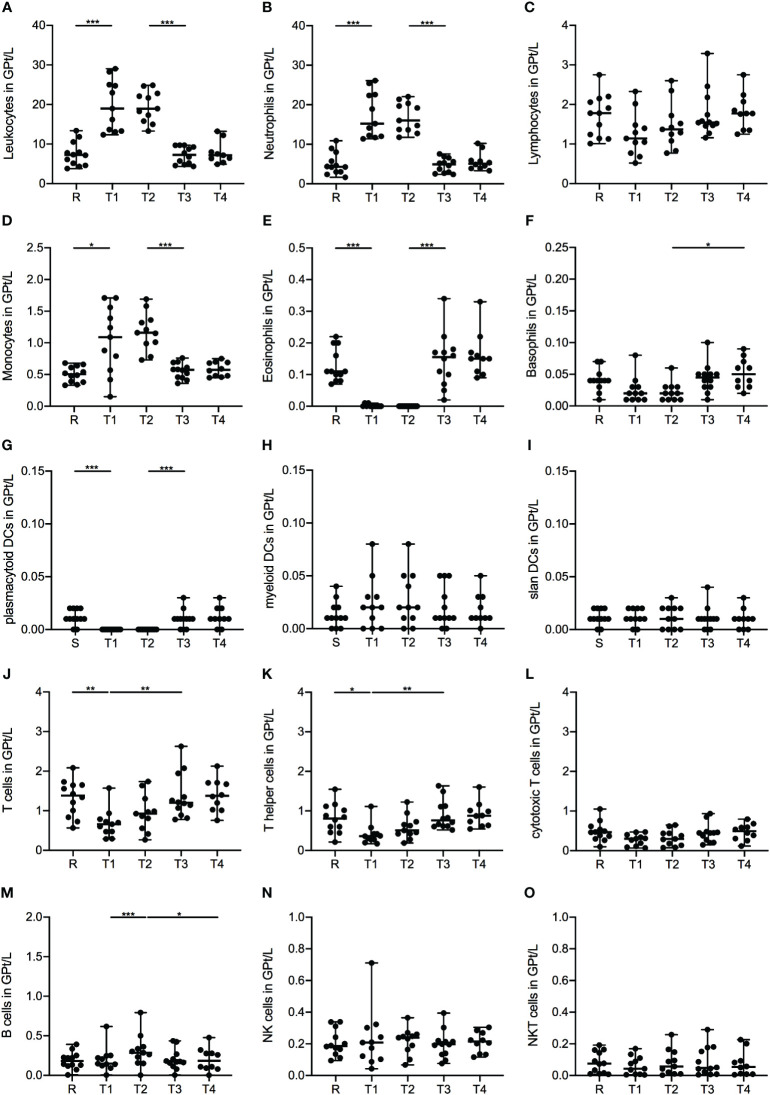
Impact of 1g/day intravenous methylprednisolone treatment on the cell count of peripheral immune cell subsets in treatment naïve patients (*n*=12). Absolute cell counts are shown at relapse (R), 24 hours after 1^st^ Infusion (T1), 24 hours after 2^nd^ Infusion (T2), two weeks (T3) and two months (T4) post-treatment. Mean values and standard deviations are revealed for leukocytes **(A)**, neutrophil granulocytes **(B)**, lymphocytes **(C)**, monocytes **(D)**, eosinophil granulocytes **(E)**, basophil granulocytes **(F)**, plasmacytoid dendritic cells (BDCA4^+^CD123^+^) **(G)**, myeloid dendritic cells (BDCA3^+^HLADR^+^) **(H)**, slan dendritic cells (MDC8^+^HLADR^+^) **(I)**, T cells (CD3^+^) **(J)**, T helper cells (CD3^+^CD4^+^) **(K)**, cytotoxic T cells (CD3^+^CD8^+^) **(L)**, B cells (CD19^+^CD3^-^) **(M)**, natural killer cells (CD3^-^CD56^+^) **(N)** and natural killer T cells (CD3^+^CD56^+^) **(O)**. Asterisks indicate statistically significant differences (* p ≤ 0.05, ** p ≤ 0.01, *** p ≤ 0.001).

Treatment with corticosteroids was associated with a significant increase in leukocytes defined by increased counts of neutrophil granulocytes and monocytes after the first MP infusion (R-T1), while lymphocyte counts were not significantly affected (**Figures 1A–D**). Further subdivision of the monocytes revealed that these changes were primarily attributed to a significant expansion of classical monocytes (CD14^+^CD16^-^), while the intermediate (CD14^+^CD16^+^) and non-classical (CD14^-^CD16^+^) subsets remained unaltered (data not shown). Eosinophils exhibited a pronounced decrease during IVMP treatment (R-T1), whereas basophils decreased only slightly without statistical significance (**Figures 1E, F**). While pDCs (BDCA4^+^CD123^+^) showed a significant decline during corticosteroid treatment (R-T1), myeloid DCs (mDCs, BDCA3^+^HLADR^+^) and 6-sulfo LacNAc1 (slan)DC counts (MDC8^+^HLADR^+^) were not significantly affected (**Figures 1G–I**).

Analysis of lymphocyte subsets presented a significant decrease in the frequency of T cells (CD3^+^) during corticosteroid treatment (R-T1), primarily driven by a decline in the T-helper (Th) cell (CD4^+^) fraction (**Figures 1J, K**), especially within the memory Th cell (CD4^+^CD45RO^+^) subset (**Table 2**). However, cytotoxic T cells (CD8^+^) showed no notable alterations during the observation period (**Figure 1L**). Further analysis of the T cell subset revealed higher frequencies of the naïve phenotype (CD45RA^+^) within the CD8^+^ subset, while the CD4^+^ fraction was dominated by a memory phenotype. Interestingly, for both T cell fractions, CD4^+^ and CD8^+^, we did not observe significant changes in the ratio of naïve and memory cells during IVMP therapy (**Table 2**). Additionally, we observed a non-significant decreasing trend in Th1 cells during corticosteroid treatment (R-T1), while no differences were noted in Th17 cell counts (**Table 2**). Analysis of the regulatory T cell (Treg, CD25^+^FOXP3^+^) subset revealed a slight decline in cell counts after the first MP infusion (R-T1) (**Table 2**). However, the ratio between Tregs an Th17 cells showed no significant alteration during the observation period.

**Table 2:** Peripheral blood lymphocyte subpopulation cell counts at relapse (R), 24h after the 1^st^ infusion of 1g methylprednisolone (T1), 24h after the 2^nd^ infusion (T2) and two weeks (T3) and two months (T4) post-treatment.

	Relapse	T1	T2	T3	T4
T cells (GPt/L)					
CD4^+^ naïve	0.277 ± 0.051	0.168 ± 0.039	0.164 ± 0.033	0.282 ± 0.033	0.282 ± 0.060
CD4^+^ memory	0.447 ± 0.062	0.207 ± 0.042*	0.369 ± 0.075	0.514 ± 0.084	0.544 ± 0.102
CD8^+^ naïve	0.277 ± 0.060	0.177 ± 0.033	0.180 ± 0.031	0.214 ± 0.035	0.200 ± 0.033
CD8^+^ memory	0.153 ± 0.025	0.075 ± 0.019	0.108 ± 0.030	0.166 ± 0.036	0.214 ± 0.044
Treg	0.030 ± 0.004	0.014 ± 0.003*	0.019 ± 0.003	0.031 ± 0.003	0.028 ± 0.005
Th1	0.454 ± 0.069	0.391 ± 0.107	0.383 ± 0.069	0.545 ± 0.106	0.584 ± 0.074
Th17	0.0014 ± 0.0003	0.0010 ± 0.0002	0.0014 ± 0.0003	0.0018 ± 0.0005	0.0013 ± 0.0002
B cells (GPt/L)					
naïve	0.22 ± 0.05	0.21± 0.07	0.204 ± 0.051	0.222 ± 0.063	0.207 ± 0.064
memory	0.10 ± 0.03	0.07 ± 0.02	0.080 ± 0.021	0.090 ± 0.032	0.102 ± 0.027
Breg	0.02 ± 0.01	0.03 ± 0.01	0.043 ± 0.012	0.020 ± 0.005	0.026 ± 0.011
Plasmablast	0.03 ± 0.01	0.03 ± 0.01	0.032 ± 0.009	0.021 ± 0.004	0.024 ± 0.006
Plasmacells	0.03 ± 0.01	0.02 ± 0.00	0.020 ± 0.005	0.020 ± 0.003	0.025 ± 0.005
NK cells (GPt/L)					
CD56^++^CD16^low^	0.02 ± 0.00	0.01 ± 0.02	0.03 ± 0.02	0.01 ± 0.00	0.01± 0.00
CD56^+^CD16^low^	0.08 ± 0.01	0.11 ± 0.02	0.19 ± 0.03**	0.08 ± 0.01	0.09 ± 0.01
CD56^+^CD16^high^	0.16 ± 0.02	0.19 ± 0.05	0.13 ± 0.02	0.19 ± 0.03	0.16 ± 0.01
NK cell HLADR^+^	0.02 ± 0.00	0.10 ± 0.02*	0.21 ± 0.05**	0.03 ± 0.01	0.02 ± 0.00
NK cell CD69^+^	0.11 ± 0.02	0.19 ± 0.04	0.24 ± 0.04	0.11 ± 0.02	0.09 ± 0.01
NK cell CD94^+^	0.14 ± 0.02	0.11 ± 0.02	0.09 ± 0.02	0.14± 0.02	0.13 ± 0.01

Breg, regulatory B cell; CD, cluster of differentiation; g, gram; GPt/L, giga-particles per liter; h, hours; HLADR, Human Leukocyte Antigen-DR isotype; NK cells, Natural Killer cells; Th, T helper cell; Treg, regulatory T cell.

Values are given as mean ± standard deviation.

Changes compared to baseline (relapse) * p ≤ 0.05, ** p ≤ 0.01.

Examination of total B cell counts (CD19^+^) during corticosteroid treatment showed an increase in peripheral CD19+-cells following the administration of the second IVMP infusion (**Figure 1M**). No significant changes were found in the frequencies of distinct B cell subsets. Our analysis included naïve and memory B cells, regulatory B cells (Breg), plasmablasts and plasmacells (**Table 2**).

Regarding the NK cell population (CD3^-^CD56^+^) and their activating and inhibitory receptors, we found that only the subset of the CD56^dim^CD16^low^, a predominantly cytotoxic fraction, was significantly increased 24 hours after the second MP administration (R-T2) (**Figure 1N**, **Table 2**). The expression of activation markers, such as very early activation antigen CD69 and Human Leukocyte Antigen DR-Isotype (HLA-DR), on the surface of NK cells, expanded during corticosteroid treatment (**Table 2**). No significant alterations were observed for the inhibitory surface receptor CD94 (**Table 2**) and the NKT cell fraction (CD3^+^CD56^+^) (**Figure 1O**).

Two weeks after completing corticosteroid treatment, the cell counts of all immune subsets returned to pre-treatment levels, with no further changes noted at the two-month post-treatment time point.

### 3.3 Different effects of corticosteroid treatment on peripheral immune cell subsets in selected DMT groups

In the second part of this study, we aimed to evaluate whether long-term immunomodulatory treatment with either a PT or FTY was associated with alterations in the response to corticosteroid treatment compared to patients wDMT.

**Figure 2:**
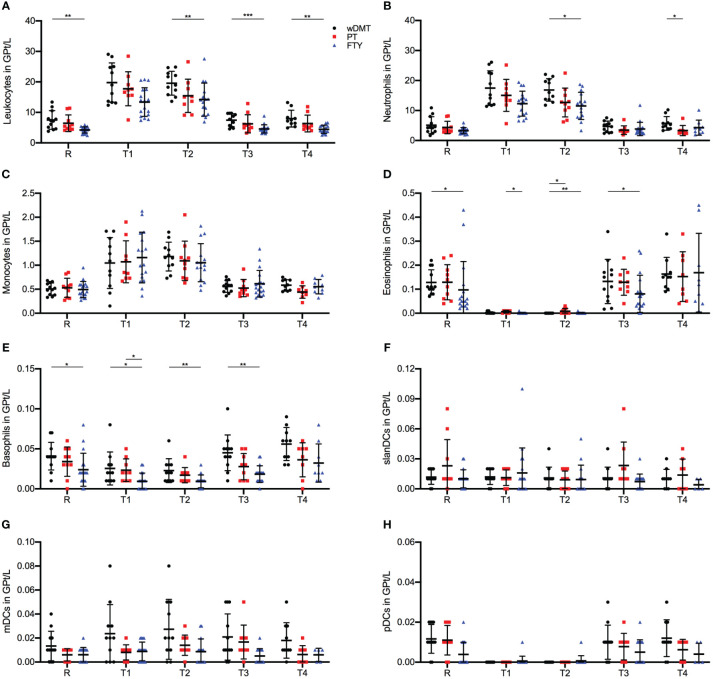
Impact of 1g/day intravenous methylprednisolone treatment on the cell count of peripheral immune cell subsets in treatment naïve patients (•, *n*=12) compared to patients treated with a platform therapy (**□**, *n*=10) or fingolimod (Δ, *n*=18). Platform therapies include Peginterferon beta-1a, Dimethyl fumarate and Teriflunomide treatment. Absolute cell counts are shown at relapse (R), 24 hours after 1^st^ Infusion (T1), 24 hours after 2^nd^ Infusion (T2), two weeks (T3) and two months (T4) post-treatment. Mean values and standard deviations are revealed for leukocytes **(A)**, neutrophil granulocytes **(B)**, monocytes **(C)**, eosinophil granulocytes **(D)**, basophil granulocytes **(E)**, slan dendritic cells (MDC8^+^HLADR^+^) **(F)**, myeloid dendritic cells (BDCA3^+^HLADR^+^) **(G)** and plasmacytoid dendritic cells (BDCA4^+^CD123^+^) **(H)**. Asterisks indicate statistically significant differences (* p ≤ 0.05, ** p ≤ 0.01, *** p ≤ 0.001).

Across all three treatment groups, leukocyte, neutrophil and monocyte cells exhibited a significant increase in frequencies following the first IVMP infusion (R-T1 p ≤ 0.001 for wDMT, PT and FTY, **Figures 2A–C**). Leukocytes counts were consistently highest in the wDMT group and lowest in the FTY group (**Figure 2A**), whereas monocytes showed no substantial differences in frequencies between the study groups during the observation period (**Figure 2C**). In contrast, eosinophils demonstrated a decline in cell counts in all three groups immediately after the first IVMP administration (R-T1 p ≤ 0.001 for wDMT and PT, and p = 0.008 for FTY), with only minor differences between the cell counts of the three groups (**Figure 2D**). For basophils, only the FTY group exhibited a slight, but statistically significant, reduction of cells during IVMP (R-T1 p = 0.024 for FTY), while the cell counts of patients wDMT and PT remained unchanged. Comparing the treatment groups indicated significantly higher frequencies of basophil cells in the wDMT cohort compared to the FTY cohort, except at timepoint T4, where no significant difference was observed (**Figure 2E**). Regarding DCs, only pDC counts declined in all three treatment groups during IVMP (R-T1 p ≤ 0.001 for wDMT and PT, and p = 0.034 for FTY), without significant differences between the three cohorts in the response of pDCs to MP treatment (**Figure 2H**). The analysis of slanDCs and mDCs revealed no changes during IVMP in any of the three groups (**Figures 2F, G**). Apart from basophils, no significant differences were detected in the response of innate immune cells to corticosteroid therapy among the three therapy groups. So generally speaking, an IVMP has the same effect on innate immune cells of pwMS, regardless of the DMT (wDMT, PT or FTY) taken.

**Figure 3:**
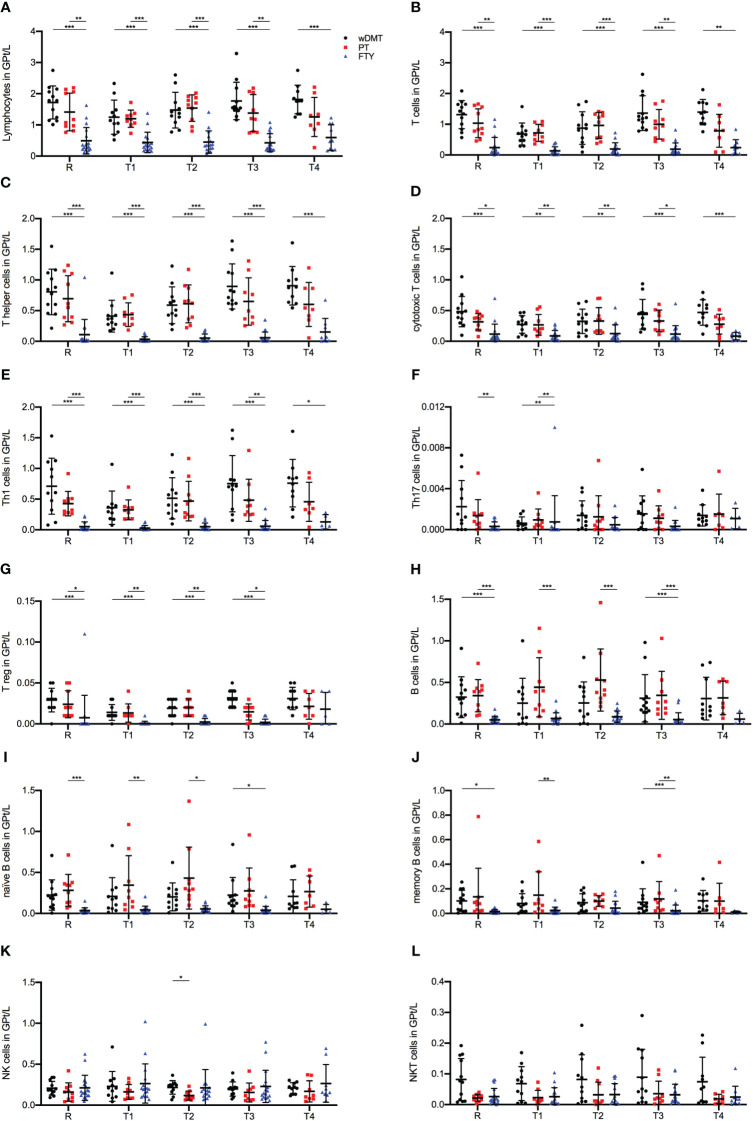
Impact of 1g/day intravenous methylprednisolone treatment on the cell count of lymphocyte subsets in treatment naïve patients (•, *n*=12) compared to patients treated with a platform therapy (**□**, *n*=10) or fingolimod (Δ, *n*=18). Platform therapies include Peginterferon beta-1a, Dimethyl fumarate and Teriflunomide treatment. Absolute cell counts are shown at relapse (R), 24 hours after 1^st^ Infusion (T1), 24 hours after 2^nd^ Infusion (T2), two weeks (T3) and two months (T4) post-treatment. Mean values and standard deviations are revealed for lymphocytes **(A)**, T cells (CD3^+^) **(B)**, T helper cells (CD3^+^CD4^+^) **(C)**, cytotoxic T cells (CD3^+^CD8^+^) **(D)**, Th1 cells (INF^+^CD3^+^CD4^+^) **(E)**, Th17 cells (IL-17^+^CD3^+^CD4^+^) **(F)**, regulatory T cells (CD25^+^FOXP3^+^CD3^+^CD4^+^) **(G)**, B cells (CD19^+^CD3^-^) **(H)**, naïve B cells (CD19^+^CD3^+^CD27^-^) **(I)**, memory B cells (CD19^+^CD3^-^CD27^+^) **(J)**, natural killer cells (CD3^-^CD56^+^) **(K)** and natural killer T cells (CD3^+^CD56^+^) **(L)**. Asterisks indicate statistically significant differences (* p ≤ 0.05, ** p ≤ 0.01, *** p ≤ 0.001).

When comparing lymphocyte cell counts among the three study groups, we identified significant differences in the response to an IVMP. Across all T and B lymphocyte populations studied, the FTY cohort displayed statistically significantly lower cell counts compared to the wDMT and PT cohorts during the whole observation period (**Figures 3A–J**). This discrepancy can be attributed to FTY-induced sequestration of lymphocytes, mainly naïve T cells. To gain a comprehensive view of lymphocyte composition, in addition to absolute cell counts, we examined the relative proportions of lymphocyte subpopulations (**Supplementary Figure 1**). Notably, T cells constituted the majority of lymphocytes in patients wDMT and PT, while patients with FTY primarily exhibited NK cells as the dominant lymphocyte population (**Supplementary Figures 1B, H, K**).

For absolute T cell counts, we observed a significant decline during IVMP in patients wDMT (R-T1 p = 0.005), while the PT and FTY cohorts did not exhibit significant changes. Throughout all time points, patients wDMT displayed the highest T cell counts, followed by patients on PT (**Figure 3B**). The cytotoxic subpopulation of T cells showed the same distribution of lymphocyte frequencies among the study groups as described above, but with no notable change during IVMP in either group (**Figure 3D**). The most substantial decline in cell counts during IVMP was observed within the Th cell population in the wDMT cohort (R-T1 p = 0.035). PT, however, experienced a less extensive decline (R-T1) with no statistical significance, resulting in an alignment of Th cell counts in patients wDMT and PT during MP treatment. FTY-treated patients started with an already preexisting low cell count before IVMP (R) and did not show a further significant decline (**Figure 3E**). Subdivision of Th cells revealed that the majority of Th cells across all cohorts were Interferon-γ producing Th1 cells, with Th17 cells and Tregs representing only a minor proportion (**Supplementary Figures 1E–G**). The FTY group exhibited the largest proportion of Tregs (**Supplementary Figure 1G**), yet absolute numbers indicated that FTY had the lowest cell count for Tregs as well as Th1 and Th17 populations (**Figures 3E–G**). Among the three cohorts, only the wDMT group showed a slight decrease in cell counts in Tregs during IVMP (R-T1 p = 0.044 for wDMT).

B cells counts at relapse (R) were highest in the PT group and significantly lower in FTY-treated patients. Following the second administration of MP, we observed a significant increase in cell frequencies by 90% in patients with PT compared to baseline levels (R-T2 p ≤ 0.001 for PT). However, B cells from the FTY cohort remained unaltered during corticosteroid treatment (**Figure 3H**). Further subdivision revealed that the increase of B cells mainly stemmed from the expansion of naïve B cells, while memory cells counts remained stable during IVMP in all three groups (**Figures 3I, J**).

Regarding NK cells, there were no observable impacts of a corticosteroid treatment on NK and NKT cells counts in either treatment group. Only at timepoint T2 were cell frequencies significantly higher in patients wDMT compared to patients with PT (**Figures 3K, L**).

## 4 Discussion

We conducted detailed immunophenotyping during corticosteroid treatment in pwMS with distinct treatment strategies experiencing a relapse. GCs exert functions on nearly all nucleated cells, though the effects vary among different cell types (20). So far, studies have primarily focused on the role of CD4 T cells. Only recently has attention shifted towards exploring other lymphocyte subtypes and innate immune cells (21, 22). Therefore, in our study, we conducted a comprehensive analysis of cells from both the innate and adaptive immune systems.

For the cells of the innate immunity, we observed an increase in neutrophilic granulocytes across all three treatment groups. Previous reports on GCs attributed those findings to an augmented release of neutrophils from the bone marrow and the inhibition of apoptosis (23). However, despite neutrophilia in blood, the simultaneous reduction in adhesion of these cells to endothelium suggests a diminished ability of neutrophils to cross into the CNS (24). A potential reduction of neutrophils within the CNS along with the simultaneous decrease in the production of proinflammatory mediators, might contribute to the therapeutic effect of GCs in relapse therapy for MS (23).

Additionally, we noted a significant increase in classical monocytes during IVMP in all three groups, consistent with previous reports (9, 25). Further functional investigations in MS patients during IVMP revealed a shift towards an anti-inflammatory M2 monocyte phenotype believed to aid in resolving neuroinflammation (9, 26).

Consistent with previous reports, eosinophil granulocytes and basophils experienced a significant drop in cell counts during GC therapy (17). Studies on allergic diseases have shown that GCs lead to increased apoptosis of eosinophils, which is explained by the inhibition of survival factors (27, 28). The role of eosinophils and basophils in MS is still not fully understood, but one could speculate that the release of cytotoxic granula contributes to neuroinflammation, as observed in other autoimmune disease (29). IVMP may reduce the proinflammatory characteristics of both cells, but concurrently elevate the risk of parasitic infections, given their importance as effector cells in such infections (27, 30).

Among DC subsets, only pDCs showed significant alterations following IVMP across all treatment groups. In a previous study, we reported that the percentage of slanDCs decreased after IVMP therapy at relapse, a finding that we could not confirm in this study (31). Due to the different methods and staining of the cells there is only limited comparability of the results. The decrease in pDCs after IVMP seems to result from the induction of apoptosis and a lack of differentiation of immature DCs, leading to reduced antigen presentation to T cells (32–34). This reduction might result in decreased activation and restimulation of T cells in the CNS.

Conflicting results exists regarding the effect of GCs on NK cell frequencies in blood (35, 36). Our study in MS patients revealed an increase in cell counts of cytotoxic and activated NK cell subsets. However, further existing studies on functional analysis demonstrated an inhibition of NK cell effector functions, including the cytolytic function. This inhibition might be due to the decreased expression of adhesion molecules to target cells and the inhibition of effector molecules perforin and granzyme A and B (35, 36).

Consistent with data from other studies, the primary alteration of cell frequencies during IVMP within the adaptive immune system was notable in CD4^+^ Th cells among patients wDMT. Due to the retention of lymphocytes in the lymph node, patients treated with FTY exhibited significantly lower T cell counts across all T cell subsets compared to patients wDMT and PT. In MS, Th1 and Th17 cells are considered the primary pathogenic subsets, with increased frequencies observed in the blood and CSF of pwMS (1, 37). Th1 cells have been demonstrated to be sensitive for GC-induced apoptosis (6, 20). Interestingly only patients wDMT displayed a reduction in cell counts, which reached no statistical significance. Regarding the susceptibility of Th17 cells to a GC treatment, conflicting results exist (38, 39). Most studies indicate that Th17 cells exhibit resistance to GC-induced apoptosis, a finding consistent with our study, where no significant alteration in Th17 cell frequencies was noted across all treatment groups following IVMP. This resistance appears to be associated with the expression of the apoptosis regulator B-cell lymphoma 2 (BCL-2) and may also depend on the concentration of IL-6 present (38–40). In contrast to other reports on Tregs, we noticed a reduction in cell counts after IVMP. Studies conducting a 5-day IVMP treatment reported an increase in Treg counts after GC therapy, attributed to the upregulation of the transcription factor FoxP3 (41, 42). As a Th cell subset, we interpret the short-term reduction in Tregs in our study within the context of the general decline in Th cells after the first MP infusion.

Since the introduction of B cell-depleting therapies, the role of B cells in MS has come more into focus (43). While showing sensitivity to GC treatment, B cells display this effect to a lesser extent compared to Th cells. Notably, immature B cells appear to be more affected than mature B cells (20). While general studies on GCs describe a GC-induced apoptosis in B cells, relatively few studies have been conducted in pwMS (6, 20). Our results, as well as investigations of Gallo et al., have indicated an increase in absolute B cell counts among patients wDMT and PT during IVMP treatment, particularly in the naïve B-cell subpopulation (44). It remains unclear whether the rise in cell counts can be explained by an enhanced release of cells to the peripheral blood, potentially accounting for the predominant effect on naïve B cells, or by alterations in the migratory properties of these cells. However, B cell subsets as well as total B cells of patients wDMT and FTY did not show any alterations during IVMP. The impact of GCs on B cells seems to depend on various factors, such as the differentiation stage and cytokine milieu, which might explain the broad spectrum of effects described in studies (20, 45). We did not analyze the effects of IVMP treatment in pwMS receiving B cell-depleting therapies, but FTY treatment is also associated with effects on B cell counts as it inhibits the egress of cells from the lymph nodes. Based on the limited effects of an GC treatment on the B cell subsets, one could speculate that the interaction of B cells with T cells is most likely to contribute to the therapeutic effect rather than the reduction of B cell numbers.

Different reports discuss insufficient clinical response in up to 40% of treated patients and differences in GC response on peripheral immune cells in MS patients versus healthy controls (46, 47). In our study, the most pronounced changes on immune cells were detected in the cohort of DMT-naïve patients. Interestingly, we did not observe a significant further reduction in T cells during IVMP treatment among patients with a pre-existing lymphopenia under FTY therapy. Patients treated with PT exhibited only a minor, yet also not significant, reduction in T cells. Given that DMTs primarily target the adaptive immune system, it can be assumed that immunomodulatory pre-treated immune cells might exist in a distinct activation state, making them less susceptible to IVMP (6, 7). These observations raise the question of whether an IVMP therapy has the same efficiency across patients with different DMTs and how distinct T cell subsets including Th17 cells drive steroid-resistant inflammation (48, 49). In our study cohort, the majority of patients across all treatment groups reported either good or partial therapeutic benefits after IVMP. Within the FTY cohort, most patients experienced complete symptom improvement, while in the wDMT and PT groups, many reported a partial response. As the number of patients without symptom improvement was too small within the groups, further subdivision between responders and non-responders was not feasible and no immune cell patterns could be derived from our data (**Supplementary Table 1**). Therefore, additional studies are necessary to investigate the potential of immunophenotyping and functional analysis of cells as a biomarker for assessing patient responsiveness to IVMP therapy.

Our findings align with existing reports, indicating that T cell apoptosis is not a mandatory requirement for the therapeutic effect of GCs. Even in mice, refractory to T cell apoptosis, therapeutic effects can still be observed (50). This implies that other immune regulatory mechanisms, such as alterations in cell migration, restauration of the blood-brain barrier, reduction of cerebral edema, and promotion of an anti-inflammatory orientation within the innate immune cells, cytokines and chemokines may play more pivotal roles in resolving neuroinflammation than previously presumed (6, 20, 51). Furthermore, this raises the question of whether MS relapse is primarily driven by T cells, as previously believed, or if pathogenic mechanisms, like the imbalance of various immune cells, contribute to the exacerbation of disease activity (1).

However, it is important to consider certain factors when interpreting our results. Even among therapy-naïve patients, we observed highly interindividual responses of immune cell populations to GC treatment. These variations become evident due to the wide dispersion around the mean during IVMP, particularly within the T lymphocyte population. The pronounced interindividual variability, which can be attributed to both genetic factors, such as polymorphisms, and environmental factors, contributes to a high level of heterogeneity in the data of existing studies (1, 52). Consequently, drawing universally applicable conclusions proves challenging.

Another consideration in the search of immune targets is, that many immune cells do not exclusively possess pro- or anti-inflammatory properties as they often have effector functions in both directions. This duality is exemplified in experiments with knock-out mice, where the complete elimination of CD8^+^ or NK cells –both assumed to be involved in MS immunopathogenesis- resulted in disease exacerbation (53, 54).

In our study, all affected cell populations exhibited either an increase or decrease in frequencies 24 hours after the first infusion, returning to pre-treatment levels two weeks after therapy. No significant alterations were noted between the timepoints before MP administration and two weeks or two months after IVMP. This observation strengthens the notion, based on the immunological changes in the blood, that a second cortisone treatment cycle might be indicated in case of persistent clinical symptoms after a two-week interval as our findings suggest transient effects of a IVMP (55). In addition, these findings indicate that a possible increased risk of infection during MP therapy, could be completely reversed after two weeks post-treatment. Furthermore, the rapid normalization of cell counts underscores that high-dose IVMP is the preferred treatment of acute disease activity but has no significant intermediate to long-term effect on the peripheral immune cells studied. Our results align with other studies that also did not find lasting changes within a period of six-month after IVMP treatment (56). In line with the immunological data, clinical data for optic neuritis have also shown no long-term clinical benefit from a MP therapy regarding functional outcomes or subsequent attack frequencies (57, 58). Consequently, our results affirm the importance of DMT for addressing enduring immunological changes that can prevent future relapses.

However, due to the limited number of patients in our treatment groups, caution must be exercised when interpreting our data. The partially strong scattering of the data points results in wide standard deviations, raising the possibility that significant differences may not have been detected when comparing means. In search of an easily accessible biomarker, we conducted immunophenotyping on peripheral cells. However, it is important to note, that the processes occurring in the blood provide only limited insights into the mechanisms of action of MP on immune cells in the CNS, which is the actual site of neuroinflammation. Furthermore, our primary focus of this work was on immunophenotyping of cells, which means that statements about their functionality are only indirectly possible, since mechanistic analyses were not part of our investigation. To gain a deeper insight in the immunopathogenesis during relapse, future studies with larger sample sizes should consider including CSF analysis.

In conclusion, in addition to T cell suppression, it appears that other immunoregulatory mechanisms of GCs are of greater significance than previously understood. Therefore, further research aimed at identifying treatment targets should broaden its scope beyond the immunomodulation of lymphocytes. It should encompass exploration of cells of the innate immune system, cell crosstalk, as well as chemokines and cytokines. Given the substantial variations among treatment groups in our study and considering the interindividual variability in immune responses, applying immunophenotyping to a general MS cohort seems less viable. Larger studies with DMT subgroups are necessary to ascertain whether immunophenotyping could serve as a biomarker for GC therapy response. Moreover, the observed variations underscore the importance of personalized treatment decisions, which will be imperative in the future.

## Data availability statement

The original contributions presented in the study are included in the article/**Supplementary Material**. Further inquiries can be directed to the corresponding author.

## Ethics statement

The studies involving humans were approved by Institutional review board of the University Hospital of Dresden (Ethikkommission an der Technischen Universität Dresden). The studies were conducted in accordance with the local legislation and institutional requirements. The participants provided their written informed consent to participate in this study.

## Author contributions

LH: Writing – review & editing, Writing – original draft. UP: Writing – review & editing. HI: Writing – review & editing. TZ: Writing – review & editing. KA: Writing – review & editing.
