# Supplementary material for: Corticosteroid-depending effects on peripheral immune cell subsets vary according to disease modifying strategies in multiple sclerosis
Source: Front Immunol. 2024 Jun 13;15:1404316. doi: 10.3389/fimmu.2024.1404316 (PMC11208457; doi:10.3389/fimmu.2024.1404316)
Supplement: Supplementary file 1 [file DataSheet_1.pdf]

Supplementary Material

Corticosteroid-depending effects on peripheral immune cell subsets vary according to disease modifying strategies in multiple sclerosis

Lena Höpner, Undine Proschmann, Hernan Inojosa, Tjalf Ziemssen, Katja Akgün*

Center of Clinical Neuroscience, Department of Neurology, Faculty of Medicine and University Hospital Carl Gustav Carus, Technical University Dresden, Dresden, Germany

*** Correspondence:**
Katja Akgün
Katja.Akguen@uniklinikum-dresden.de

1 Supplementary Figures Legend

Supplementary Figure 1. Impact of 1g/day intravenous methylprednisolone treatment on peripheral immune cell subsets in treatment naïve patients (•, *n*=12) compared to patients treated with a platform therapy (□, *n*=10) or fingolimod (△, *n*=18). Platform therapies include Peginterferon beta-1a, Dimethyl fumarate and Teriflunomide treatment. Relative proportions are shown at relapse (R), 24 hours after 1st Infusion (T1), 24 hours after 2nd Infusion (T2), two weeks (T3) and two months (T4) post-treatment. Mean values and standard deviations are revealed for leukocytes in GPt/L (A), neutrophil granulocytes % of leukocytes (B), monocytes % of leukocytes (C), eosinophil granulocytes % of leukocytes (D), basophil granulocytes % of leukocytes (E), 6-sulfo LacNAc1 (slan) dendritic cells % peripheral blood mononuclear cells (PBMC) (F), myeloid dendritic cells % PBMC (G), plasmacytoid dendritic cells % of PBMC (H). Asterisks indicate statistically significant differences (* $p \leq 0.05$, ** $p \leq 0.01$, *** $p \leq 0.001$).

Supplementary Figure 2. Impact of 1g/day intravenous methylprednisolone treatment on the cell count of lymphocyte subsets in treatment naïve patients (•, *n*=12) compared to patients treated with a

platform therapy (□, n=10) or fingolimod (△, n=18). Platform therapies include Peginterferon beta-1a, Dimethyl fumarate and Teriflunomide treatment. Relative proportions are shown at relapse (R), 24 hours after 1st Infusion (T1), 24 hours after 2nd Infusion (T2), two weeks (T3) and two months (T4) post-treatment. Mean values and standard deviations are revealed for lymphocytes % of leukocytes (A), T cells % of lymphocytes ($CD3^+$) (B), T helper (Th) cells ($CD3^+CD4^+$) % of lymphocytes (C), cytotoxic T cells ($CD3^+CD8^+$) % of lymphocytes (D), Th1 cells (INF $\gamma^+CD3^+CD4^+$) % of Th cells (E), Th17 cells ($IL\text{-}17^+CD3^+CD4^+$) % of Th cells (F), regulatory T cells ($CD25^+FOXP3^+CD3^+CD4^+$) % of Th cells (G), B cells ($CD19^+CD3^-$) % of lymphocytes (H), naïve B cells ($CD19^+CD3^+CD27^-$) % of B cells (I), memory B cells ($CD19^+CD3^-CD27^+$) % of B cells (J), natural killer cells ($CD3^-CD56^+$) % of lymphocytes (K) and natural killer T cells ($CD3^+CD56^+$) % of lymphocytes (L). Asterisks indicate statistically significant differences (* $p \leq 0.05$, ** $p \leq 0.01$, *** $p \leq 0.001$).

2 Supplementary Table

Supplementary Table 1. Peripheral blood Th17 cell count in responders versus non-responders to intravenous methylprednisolone treatment in patients with multiple sclerosis.

**Th17 cell count (GPt/L)**	**All** (*n*=40)	**Responder**[2] (*n*=31)	**Non-Responder**[2] (*n*=9)
**wDMT**	(n=12)	(n=10)	(n=2)
R	0,0022 ± 0,0024	0,0022 ± 0,0026	0,0015 ± 0,0015
T1	0,0006 ± 0,0006	0,0007 ± 0,0006	0,0003 ± 0,0003
T2	0,0014 ± 0,0014	0,0017 ± 0,0013	0,0000 ± 0,0000
T3	0,0015 ± 0,0017	0,0017 ± 0,0018	0,0007 ± 0,0007
T4	0,0014 ± 0,0010	0,0015 ± 0,0010	0,0007 ± 0,0003
**PT**[1]	(*n*=10)	(*n*=8)	(*n*=2)
R	0,0014 ± 0,0015	0,0014 ± 0,0016	0,0011 ± 0,0003
T1	0,0010 ± 0,0010	0,0011 ± 0,0011	0,0003 ± 0,0001
T2	0,0012 ± 0,0020	0,0014 ± 0,0022	0,0006 ± 0,0002
T3	0,0011 ± 0,0011	0,0013 ± 0,0011	0,0000 ± 0,0000
T4	0,0015 ± 0,0018	0,0018 ± 0,0018	0,0001 ± 0,0000
**FTY**	(*n*=18)	(*n*=13)	(*n*=5)
R	0,0003 ± 0,0004	0,0003 ± 0,0005	0,0003 ± 0,0004
T1	0,0008 ± 0,0025	0,0001 ± 0,0002	0,0025 ± 0,0043
T2	0,0005 ± 0,0007	0,0005 ± 0,0007	0,0005 ± 0,0005
T3	0,0003 ± 0,0006	0,0002 ± 0,0003	0,0006 ± 0,0009
T4	0,0011 ± 0,0009	0,0011 ± 0,0010	0,0012 ± 0,0000

FTY: fingolimod; GPt/L: Gigaparticles per litre; *n:* patient count; PT: platform therapy; Th: T-helper cell; wDMT: without disease-modifying-therapy. Time points: relapse (R), 24 hours after 1st Infusion (T1), 24 hours after 2nd Infusion (T2), two weeks (T3) and two months (T4) post-treatment. Values are given as mean ± standard deviation.

[1] platform therapy includes: Interferon (*n*=2), dimethyl fumarate (*n*=6), teriflunomide (*n*=2)

[2] subjective assessment by patients
