# Supplementary figures and images for: Corticosteroid-depending effects on peripheral immune cell subsets vary according to disease modifying strategies in multiple sclerosis
Source: Front Immunol. 2024 Jun 13;15:1404316. doi: 10.3389/fimmu.2024.1404316 (PMC11208457; doi:10.3389/fimmu.2024.1404316)

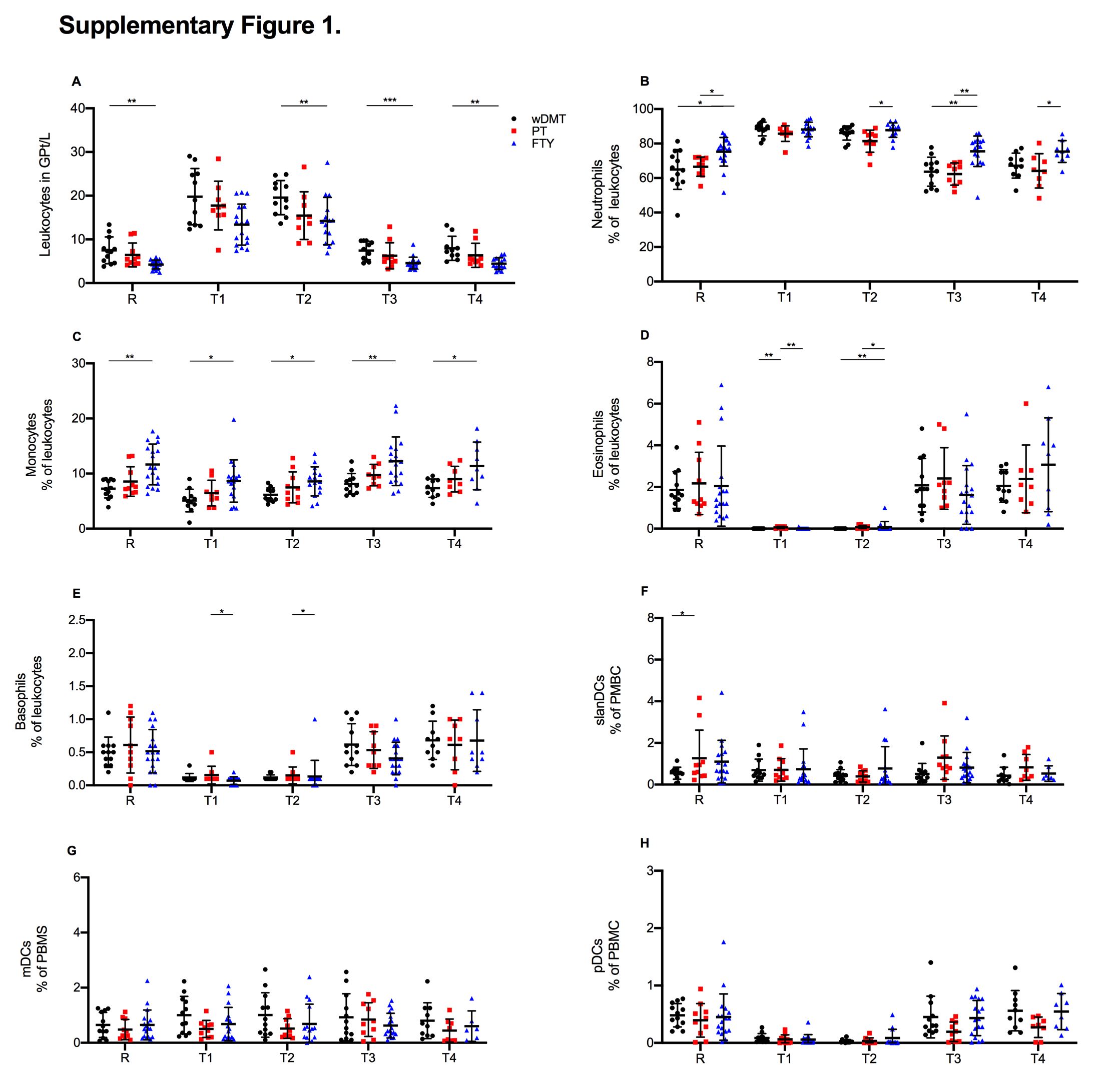

Supplement: Supplementary file 2 [file Image_1.tiff]

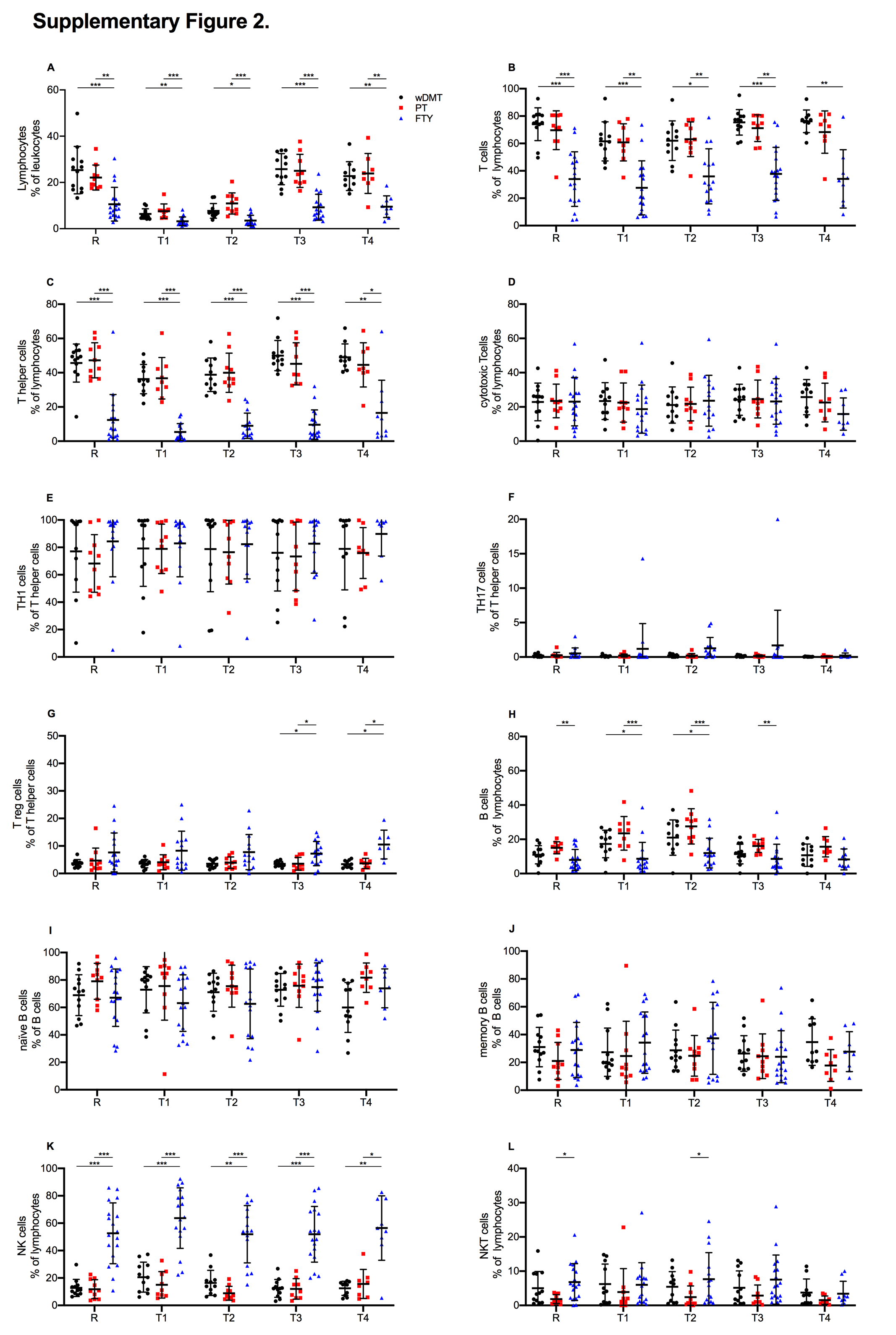

Supplement: Supplementary file 3 [file Image_2.tiff]
